# Improving our understanding of the quality of life of patients with metastatic or recurrent/persistent anal cancer: a systematic review

**DOI:** 10.1007/s00520-025-09520-8

**Published:** 2025-05-15

**Authors:** Samantha C. Sodergren, Rowan Edwards, Rahul Krishnatry, Marianne G. Guren, Kristopher Dennis, Pierfrancesco Franco, Francesca de Felice, Anne-Sophie Darlington, Vassilios Vassiliou

**Affiliations:** 1 University of Southampton, Southampton, UK; 2 Tata Memorial Centre, Homi Bhabha National Institute University, Mumbai, India; 3 Oslo University Hospital, Oslo, Norway; 4 Ottawa Hospital, Ottawa, Canada; 5 University Hospital ‘Maggiore della Carità’, Novara, Italy; 6 Sapienza University of Rome, Rome, Italy; 7 Bank of Cyprus Oncology Center, Strovolos, Cyprus; 8 University of Oslo, Oslo, Norway; 9 University of Eastern Piedmont, Novara, Italy

**Keywords:** Anal cancer, Metastatic, Recurrence, Persistence, Systemic therapy, Quality of life, Patient-reported outcomes

## Abstract

**Purpose:**

Chemoradiation (CRT) is used to treat anal carcinomas which, for most patients with loco-regional disease, results in a cure but is associated with acute and chronic complications impairing quality of life (QoL). Patients with metastatic disease or recurrence are likely to experience additional QoL concerns. This paper identifies the QoL issues of these patients and determines whether the EORTC QLQ-ANL27 (QLQ-ANL27), a measure of QoL of patients treated with CRT for anal cancer used alongside the core EORTC QLQ-C30 (QLQ-C30), is suitable or needs adapting.

**Methods:**

A systematic review was conducted of studies published between 2014 and 2024 reporting QoL of patients with metastatic or recurrent/persistent anal cancer or follow-up data of patients treated with CRT for anal cancer.

**Results:**

This review included 23 papers, only three focused exclusively on metastatic and/or recurrent anal cancer. Most of the 53 reported symptoms related to bowel, urinary, and sexual functioning, with 60% covered by the QLQ-ANL27 or the QLQ-C30. Issues not captured include, for example, neuropathy, hair loss, musculoskeletal problems, urinary incontinence, and embarrassment.

**Conclusion:**

There is a paucity of research looking specifically at QoL outcomes of patients with metastatic or recurrent anal cancer. Whilst the QLQ-ANL27 captures most QoL issues affecting these patients, it might require adapting to improve its sensitivity.

**Supplementary information:**

The online version contains supplementary material available at 10.1007/s00520-025-09520-8.

## Introduction

Anal carcinomas arise at the anal margin or within the anal canal with the vast majority being squamous cell carcinomas. These carcinomas are rare, accounting for less than 1% of all cancer diagnoses and less than 3.5% of GI cancers but their incidence is on the rise [1–3]. The current standard of care, chemoradiotherapy (CRT), achieves 5-year survival rates of 75% [4] but is not without acute and chronic toxicities significantly impacting functioning across different domains of life (i.e. physical, emotional, social, role) leading to impaired quality of life (QoL) [5]. Monitoring QoL is imperative to facilitate symptom management and contributes to safety assessments in clinical trials to support treatment protocol approvals [6, 7]. Whilst treatment safety profiles are largely informed by clinical assessments of toxicities (i.e. the Common Terminology Criteria for Adverse Events (CTCAE) [8]), it is widely accepted that QoL assessments should rely upon the patient’s perspective [9]. The European Organisation for Research and Treatment of Cancer (EORTC) Quality of Life Group (QLG) questionnaire, the EORTC QLQ-ANL27 (QLQ-ANL27), is the first validated patient reported outcome (PRO) measure specifically for patients with anal cancer treated with CRT [10, 11], designed to capture acute and chronic QoL issues. The QLQ-ANL27 supplements the core measure of the EORTC QLG, the EORTC QLQ-C30 [12] and measures bowel symptoms, pain/discomfort, urinary frequency, swelling in legs/ankles, problems with cleaning oneself and planning activities, sexual function, painful intercourse, vaginal symptoms, and erectile problems.

Whilst most patients with anal cancer present with localised disease, up to 15% are diagnosed with advanced disease, and the incidence of Phase IV disease has also grown [13]. Furthermore, 15–20% will develop disease progression after CRT for initial localised disease [14, 15]. For patients with relapsed and/or metastatic disease, other treatment options are called upon [16], including the use of platinum-based chemotherapy regimens such as carboplatin or cisplatin and paclitaxel or fluorouracil [14, 17–19], surgery, including abdominoperineal resection [20], and more recently, immunotherapies such as nivolumab, pembrolizumab, and retifanlimab [21–24] which have shown promise for patients with intolerance to platinum-based therapy or whose disease has progressed following this treatment. Patients both in the recurrent/persistent anal cancer and metastatic/advanced disease settings are therefore exposed to different side-effect profiles compared to patients with localised disease treated with CRT. In addition, for those who have been previously exposed to CRT, the long-term sequelae of CRT might still be present and further compromise their QoL. Aside from physical symptom and functioning issues, patients with advanced disease or treatment failure are likely to experience additional psychological concerns, for example regarding future uncertainty [25]. Thus, compared to patients treated with CRT alone for localised anal cancer, patients with metastatic or persistent/recurrent anal cancer are likely to experience unique and more complicated QoL concerns as a function of their disease state, including, for example affected organs, and treatment exposure which might not be covered by current QoL measures including the QLQ-ANL27.

As part of its international validation, the QLQ-ANL27 was tested with 382 patients treated with CRT, of whom six had metastatic disease, eight local recurrence, and seven locoregional recurrence [11]. It is hypothesised that whilst the QLQ-ANL27 is likely to capture many concerns experienced in the metastatic or recurrent/persistent context, the questionnaire might need to be supplemented with additional items. The EORTC QLG supports this flexible approach to measurement whereby users (e.g. researchers, clinicians) can select, from an item library, questions from the EORTC QLG’s portfolio of fully or partially validated questionnaires) to supplement existing modules and to create bespoke, customised lists [26].

The overall aim of this systematic review is to generate an exhaustive list of QoL issues relevant to patients with metastatic, recurrent, or persistent anal cancer. A secondary aim is to determine whether the QLQ-ANL27 together with the core cancer measure, QLQ-C30, covers the QoL issues extracted from the literature. This systematic review is part of a larger programme of work to improve our understanding of the QoL of patients with metastatic or recurrent/persistent anal cancer and to determine the optimal method of assessing QoL for this patient group.

## Methods

The protocol for this review (Supplementary material) was informed by the PRISMA-P statement [27].

### Search strategy

Search terms were generated following the advice of clinical experts and specialist medical librarians. Search expressions used in our previous review [5] also informed our current work. We used Boolean operator OR between each term in each area and the Boolean operator AND between each area (see Table 1). Whilst the focus of this review is on the experiences of patients with metastatic and recurrent/persistent anal cancer, it was decided to broaden the search terms to include anal cancer in general for the following reasons: (1) late effects of previous anal cancer treatments will affect this patient group and (2) some publications may refer to our patient group of interest within a broader report on all patients with anal cancer. Publications on patients successfully treated for localised anal cancer (i.e. disease-free survivors) were included where long-term QoL assessments were made at least 12 months post-treatment.

**Table 1:** Search terms

Area	Terms
Anal cancer	Anus neoplasm Anal neoplasm Anal cancer Anus cancer Anal carcinoma Anus carcinoma Anal canal cancer Anal canal carcinoma Anal tumour Anus tumour Anal intraepithelial neoplasia Anal canal intraepithelial neoplasia Anal squamous intraepithelial lesions Anal squamous cell carcinoma Anal cloacogenic carcinoma Cloacogenic carcinoma of the anal canal
Treatments	Chemoradiotherapy Radiochemotherapy Chemoradiation Chemotherapy Radiotherapy Combined modality therapy Antineoplastic chemotherapy Antineoplastic agents Colostomy Surgical stoma (Exp Stoma and stoma bag)
Quality of life	Quality of Life QOL Health related quality of life HRQOL Subjective health status Patient reported outcome Patient based outcome Patient reported outcome measure PROM Self report Side effect Toxicity Adverse effect Adverse event Safety Complication Dysfunction Disturbance Disorder Impairment Complaint Symptom

Databases were searched for English language publications dated between April 2014 (to coincide with the final time point of our previous review [5]) and November 2023. An updated search was conducted in July 2024 to capture additional eligible papers. Databases searched included MEDLINE, Psych Info, Web of Science, CINAHL, EMBASE, and the Cochrane Database of Systematic Reviews. These searches were supplemented with manual checking of selected full-text manuscripts. All available peer-reviewed literature was considered; empirical research adopting quantitative and qualitative research methods was included. Reviews, reports, and meta-analyses were considered for descriptive and cross-referencing purposes only but were not included to avoid duplication. Reports of conference proceedings, theses, abstracts, guidelines, and case reports were excluded. Studies reporting data on patients with anal cancer alongside other patient groups were excluded in case no separate reporting of QoL outcomes for anal cancer was available. For a full list of inclusion and exclusion criteria, see Table 2.

**Table 2:** Inclusion and exclusion criteria

Inclusion criteria	Exclusion criteria
Papers reporting on QoL from the perspective of patients	Papers not reporting QoL outcomes from the perspective of patients including studies where only clinician rated measures are used
Papers including patients diagnosed with anal cancer (different disease stages and follow-up points). This will include patients with localised anal cancer where long-term follow-up QoL data are provided (> 12 months) This will include studies on patients with different diagnoses alongside anal cancer, if QoL data for people with anal cancer are reported separately	Papers not providing separate data on patients diagnosed with anal cancer Papers presenting QoL data on patients during treatment for localised disease only
Papers reporting studies on adult patients aged 18 years and above	Papers reporting studies on children or adolescents diagnosed with cancer
Papers published since April 2014	Papers published before April 2014
Papers published in English Language	Non-English Language papers
Studies including the following designs: 1. Randomised controlled trials 2. Trials of quasi-experimental design (observational, case–control) 3. Qualitative studies 4. Mixed methods	Conference proceedings Theses Protocols Cost effectiveness studies Studies reporting on animals Other papers not presenting primary data (e.g. reviews, case studies, expert opinion, theoretical papers, policy documents, guidelines, consensus, letters, editorials)
Relevant grey literature from searches	

Abbreviation: QOL quality of life

### Paper selection

Database search results were imported into Endnote [X8], and duplicate records removed. All references (titles and abstracts) were subsequently transferred to Rayyan QCRI (rayyan.qcri.org), an online systematic review management platform, and a thorough screening process was conducted across two stages. In stage one, two reviewers [RE, SS] independently reviewed all title and abstracts in Rayyan against the inclusion criteria (Table 2), and any discrepancies were resolved. In the event of any remaining doubt, the full paper was obtained. During stage two, full texts of accepted papers were reviewed by RE and independently double-screened by a second reviewer [SS, MG, KD, PF, and SA] with eligibility disagreements resolved by a third reviewer.

### Data extraction and analysis

A data extraction form, adapted from the minimum data checklist [28], was created in Microsoft Excel. Data extraction was carried out by one reviewer (RE) and checked by a second reviewer [SS] and included participant characteristics (disease stage), treatments, QoL assessments, and patient reported QoL issues with particular attention to those not covered by the QLQ-ANL27 and reported in the context of metastatic or persistent/recurrent anal cancer. Toxicity data were also extracted to supplement the QoL issues reported. A descriptive synthesis was used to analyse diverse foci, disease stages, treatments, PRO measures (PROMS), and assessment periods.

The quality of publications adopting a quantitative methodology was assessed using the Efficace checklist [28], a tool for measuring QoL outcomes cancer clinical trials evaluating conceptualisation, measurement, methodology, and interpretation across 11 different indicators (maximum possible score of 11).

## Results

**Fig. 1:**
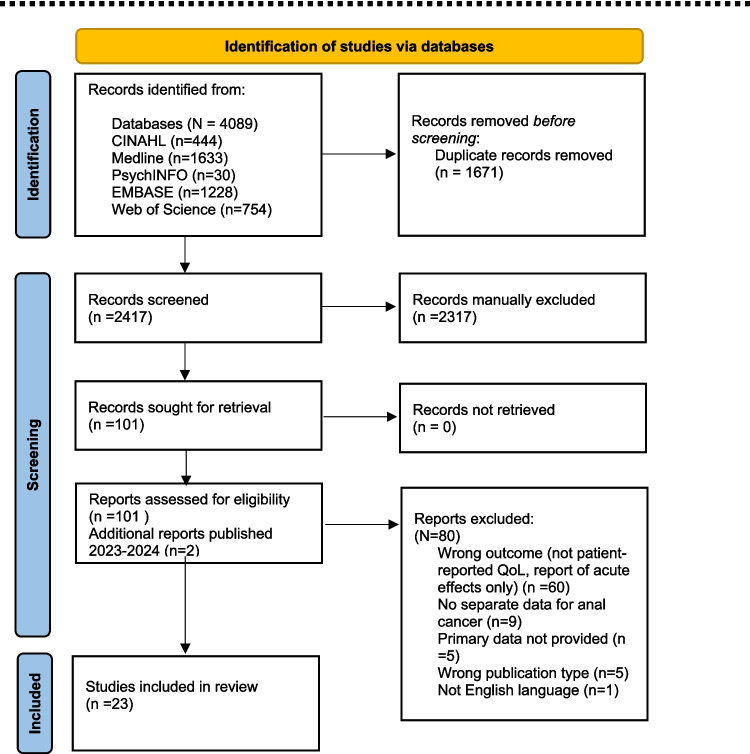
PRISMA 2020 flow diagram

The database search generated 4089 papers (Fig. 1), of which 2417 were screened and 101 full texts reviewed. Of those, a further 80 papers were excluded leaving 21 for data extraction. An additional two eligible publications were identified following an updated search of papers published up to July 2024 resulting in a total 23 included papers.

Quality assessment scores for the 22 quantitative studies ranged from 3 to 10, with a mean score of 7.86, which is lower than the score of 8.7 which Efficace et al. [28] state denotes a high-quality standard in reporting. Low scores were found for the documentation of missing data, reported in only 5 papers (23%), and baseline data provided for 8 studies (36%), due to the high proportion of retrospective studies.

Data were extracted from 23 publications (Table 3) including two with a focus on patients with metastatic disease [19, 23], one of which [19] also included those with unresectable locally recurrent disease treated with standard or modified regimens of docetaxel, cisplatin, and fluorouracil. Rao et al. [23] reported data from previously treated advanced or metastatic patients enrolled in the POD1UM trial who were receiving immunotherapy (retifanilab) and had previously been treated with radiotherapy (alone or with chemotherapy) and/or surgery including an exenteration procedure for 22% patients evaluated. A further publication [29] involved disease-free survivors following salvage surgery for recurrence. The remaining 20 studies reported data on long-term follow-up, reaching up to and beyond 6 years for some studies (e.g. [30, 31] of patients treated for cancer, of which, five [31–35] included patients with metastases and/or recurrence as part of their cohort, although a separate analysis on these patients was only provided in one study [31]. Patients participating in these studies were treated with CRT (mostly with intensity modulated radiotherapy (IMRT)) and four studies [30, 33–35] included patients who had also received surgery. Whilst eight publications (seven studies) [19, 23, 32, 34, 36–39] provided PROs for patients on more than one occasion, most were retrospective in design.

**Table 3:** Studies included in the review

First author and year	Country	Aim	Design	Sample	Treatment	Patient reported outcome measure (PROM)	Key findings	Quality assessment score**
**Studies including patients with metastatic or recurrent anal cancer treated with systemic therapies**								
**Kim (2018)** [19]	France	Clinical activity and safety of docetaxel, cisplatin, and fluorouracil in patients with metastatic or unresectable locally recurrent anal squamous cell carcinoma	Prospective phase II clinical trial. Patients assessed at study inclusion, every two cycles of chemotherapy, and after treatment and at each follow-up visit for 3 years or until death	*N* = 66 patients with metastatic disease or with unresectable local recurrence (*n* = 60 with QoL assessments)	CT: standard or modified regimens of docetaxel, cisplatin, and fluorouracil	QLQ-C30 [12]	Ninety-seven serious adverse events were recorded. The most frequent symptoms experienced include diarrhoea, fatigue, nausea, and vomiting. Other events include dysgeusia, dehydration, abdominal pain, constipation, mucositis, asthenia, peripheral neuropathy, alopecia, oedema in the limbs, hand-foot syndrome, infection, and weight loss. The global QoL favoured the modified treatment schedule	7
**Rao (2022)** [23]	UK (Europe-wide)	Safety and clinical activity of retifanlimab in patients with previously treated advanced or metastatic squamous carcinoma of the anal canal	Prospective phase II clinical trial POD1UM-202). QoL assessment carried out at screening and every cycle until cycle 5 and then before imaging assessments and at the end of treatment visit	*N* = 94 patients with locally advanced or metastatic disease 81% had distant metastases (M1) at study entry, with 70% having more than one site of metastatic disease. QoL data available at baseline for 79 and 80 patients (for QLQ-C30 and EQ-5D respectively). Approximately 30% completed the QLQ-ANL later in the study	Immunotherapy: 500-mg dose of retifanlimab every 4 weeks 87.2% had prior RT, as either CRT (73%) or RT alone (17%). Surgery: 46% had prior surgery or procedure, which included an exenteration procedure in 21 patients	QLQ-C30 [12], EQ-5D [40], QLQ-ANL27 [10, 11]	Treatment-related adverse events were reported for 55 patients and include pruritus (12%), fatigue (10%), diarrhoea (9%), asthenia (7%), nausea (6%). Fifty-one patients experienced serious adverse events including abdominal pain (5%), and urinary tract infection (4%). Most QoL scores improved with therapy, A breakdown of scores according to domain is not provided QLQ-ANL27 scores were not analysed	5
**Studies including patients successfully treated with surgery for recurrence (disease-free survivors)**								
**Pedersen (2019) **[29]	Denmark	QoL in patients with long-term disease-free survival following salvage surgery for recurrence	Retrospective Median 4 years post-surgery	*N* = 25 eligible patients (*n* = 14 returned questionnaires) Patients had undergone salvage surgery for relapse or residual tumour (disease free survivors). Median time since surgery: 4 years	Salvage surgery	QLQ-C30 [12] and QLQ-CR29 [41] and free-text response to list QoL issues not addressed in the questionnaires	The global health status and functional scores were high, whilst symptom scores were low, except for low sexual interest for women and high symptom scores for impotence for men and dyspareunia for women. All symptoms of the QLQ-C30 and CR29, were experienced by at least one respondent, most commonly fatigue (*n* = 12), dry mouth (*n* = 11), urinary frequency (*n* = 9), bloating (*n* = 9), faecal incontinence (*n* = 8), impotence (*n* = 8), buttock pain (*n* = 7). Additional issues not covered by the questionnaires were identified by 7 patients and included perineal herniation/heaviness making it difficult to sit down for long periods (*n* = 3), reduced size of penis (*n* = 1), impossibility to engage in sexual intercourse (*n* = 3), and urinary incontinence (*n* = 1)	9
**Axelsson (2022) **[30]	Sweden	Patient-reported QoL and bother 3- and 6 years post-treatment	Cross-sectional prospective cohort study 3 years and 6 years post-treatment	*N* = 195 3 years post-treatment *N* = 152 patients 6 years post-treatment. Comparison with a reference group of members of the Swedish population	CT/RT/CRT and surgery	Study-specific questionnaire of 260 items with a focus on bowel, urinary and sexual function, social and mental function, co-morbidity, lifestyle, daily activities, personal characteristics, and perceived QoL. In addition, questions on symptom severity and level of bother. Sense of coherence was also measured using the SOC-29 [42] as an explanatory variable	Low Qol at both 3 and 6 years was reported by 60% patients. Major Long-term bother from bowel (51%), urinary (33%) and sexual function (26%) with urinary function bother persisting over time. Patients experiencing bother across these domains had impaired QoL. Depression and sense of coherence could not explain dysfunction urinary and sexual function Urinary and bowel function was related to QoL	8
**Bourdais (2021) **[43]	France	Clinical outcomes and QoL of patients receiving PDRT with a boost to residual tumour after external	Retrospective study. Median follow-up 60.4 months post-treatment	*N* = 42 (*n* = 22 completed the QoL assessment)	PDR-BT boost following external beam radiotherapy (with or without CT)	Study-specific survey adapted from EORTC questions including gynaecological, urinary and bowel problems, activity limitations, fatigue, mood, and overall health perception	More than 90% of the patients reported good or very good overall quality of life. Twelve patients reported activity restrictions and nine experienced sadness. Asthenia (at grade 1 or 2) was reported by seven patients and four had grade 1 pelvic pain	3
**Chaballout (2023) **[44]	USA	To understand how patients’ diagnosis and treatment experiences affect stigma	Cross-sectional study. Median follow-up 30 months post-treatment	*N* = 46	CRT (IMRT)	Study-specific survey with questions taken from the QLQ-ANL27 [10, 11] and QLQ-CR29 [41] as well as questions about baseline knowledge and fears regarding CRT and how these matched experiences	Patients perceived short-term CRT side effects as worse than expected, however, most patients felt better off after treatment. In terms of long-term effects of treatment, bowel habit changes (73%), sexual function (65%) and interest (55%) were reported as worse than expected	9
**Corrigan (2022) **[45]	USA	Patient perceptions of QoL during and after CRT in long-term survivors of anal cancer. Describe the most prevalent themes	Retrospective cross-sectional study. Median follow-up at 50 months post-treatment	*N* = 248 (*n* = 112 completed the questionnaire, *n* = 84 answered the free-text question)	CRT (IMRT)	Study-specific questionnaire including questions about preparedness and support for late toxicities and an additional free text response to share additional thoughts about general QoL issues. FACT-G7 [46]	Most patients (70%) reported that were able to enjoy like (‘quite a bit’/’very much’). The free-text responses highlighted persistent toxicity (82%) bowel, urinary, sexual, and musculoskeletal) affecting QOL insufficient upfront information about CRT (56%), and gratitude toward care received (35%)	7
Corrigan (2022) [47]	USA	Sexual function more than 2 years after definitive CRT	Retrospective study cross-sectional study. Median follow-up 50 months post-treatment (same cohort as above)	*N = 248 (n = 112 completed the questionnaires)*	CRT (IMRT)	All patients completed the PROMIS SexFS [48]. Sexual function Men—IIEF-5 [49], Women – FSFI [50]	Patients experience significant long-term sexual dysfunction	8
**De (2022) **[51]	USA	Patient-reported bowel and urinary functional outcomes and QoL following CRT	Retrospective study cross-sectional study. Median follow-up 50 months post-treatment (Same cohort as above)	*N* = 248 (*n* = 112 completed the questionnaires)	CRT (IMRT)	FACT-G7 [46], FIQoL [52], LARS [53], ICIQ [54]	Patients experience significant long-term bowel dysfunction whilst urinary problems were relatively low. FIQoL scores were associated with higher (better) FACT-G7. Higher (worse) LARS scores were associated with lower (worse) FACT-G7 scores	8
De Francesco (2016)* [32]	UK	Long-term consequences of CRT with IMRT on bowel and sexual function	Prospective observational study. At least 12 months follow-up	*N* = 43 (*n* = 27 were available for questionnaire follow-up). Patients with metastases and recurrence included numbers not known)	CRT (IMRT)	IBDQ [55], Vaizey Incontinence Questionnaire [56], LENT SOMA [57]	IMRT affects bowel and sexual function, but late toxicity did not increase over the period observed in the study	9
Frick (2017)* [33]	USA	Late and long-term effects of gastro-intestinal cancer including anal cancer as part of the testing of an internet-based tool to create survivorship care plans	Cross-sectional study. Median 2.4 years follow-up	*N* = 1129 including colon, rectum, and anal cancer (*n* = 119) patients. Patients with metastases and recurrence included. Numbers not specified)	CT/RT/CRT/surgery/no treatment	Study-specific internet tool with questions about long-term and late effects	Patients with anal cancer had a high prevalence of sexual dysfunction and radiation-induced dermatologic effects	6
Hosni (2022)* [34]	Canada	Long-term QoL after CRT for anal cancer. Evaluation of RT dose on QoL	Prospective cohort study. Assessments at baseline, end of treatment, 3-,6-,12-months post-treatment and then annually until last follow-up. Median 56.5 months follow-up	*N* = 93 at baseline (108 enrolled). *N* = 14 patients with recurrence and *N* = 5 metastases included	CRT with IMRT, surgery	QLQ-C30 [12], QLQ-CR29 [41]	There was a long-term decline in dyspnoea, body image, bowel embarrassment, faecal incontinence, and hair loss, and long-term worsening of impotence	10
**Joseph (2016) **[36]	Canada	Prospective evaluation of the impact of IMRT on QOL during and post-treatment	Prospective Phase II clinical trial. Baseline, end of treatment, 3, 6, 12, 24, and 36 months post-treatment	*N* = 54 patients with locally advanced anal cancer	IMRT (dynamic helical- tomotherapy) based CRT	QLQ-C30 [28], QLQ-CR29 [41]	All C30 functional symptoms, except emotional and cognitive functioning, were impaired at the end of treatment and recovered by 3 months follow-up. diarrhoea, urinary incontinence, and dyspareunia persisted post-12 months	10
**Joseph (2023) **[37]	Canada	Long-term QoL and late toxicity following IMRT-CRT	Prospective Phase II clinical trial. Follow-up from Joseph (2016) with data up to 60 months post-treatment	*N* = 54	IMRT-based CRT	QLQ-C30 [12], QLQ-CR29 [41]	Whilst most patients experienced clinically significant recovery of function and improvement in QoL over 5 years post-treatment, chronic diarrhoea, faecal incontinence, urinary incontinence and dyspareunia remained significant problems	10
Knowles (2015)* [35]	UK	Prevalence of long-term urinary, bowel and sexual dysfunction and overall QoL patients following CRT	Retrospective cross-sectional study. Median follow-up 63.8 months post-treatment	*N* = 42 patients Those with recurrence were included (numbers not known) as treatment intent remained curative. Patients were disease-free at the time of the study	CRT and surgery (recurrent cases)	QLQ-C30 [12], the QLQ-CR38 [58], MSKCC Bowel function instrument [59]	Insomnia was an ongoing issue for 25% patients, 17% reported diarrhoea as ‘quite a bit’ and ‘very much’, 17% reported financial difficulties (‘quite a bit’ and ‘very much’), all of whom were under the age of 65 years Patients also reported bowel (urgency, leakage, diet and activity restrictions and flatulence) and sexual problems	8
**Koerber (2019) **[60]	Germany	Acute and chronic side effects and QoL in female patients treated with 3D-CRT or IMRT with consideration of dosimetric parameters. Develop predictors for toxicity and guide the radiotherapy planning process	Retrospective cross-sectional study. Median follow-up 3 years	*N* = 91 (*n* = 47 female patients with completed assessments)	CRT (3D and IMRT)	Study-specific questionnaire based on CTCAE [8] and LENT-SOMA [57]	Patients treated with 3D-CRT experienced greater frequency of problems relating to poor urinary stream, loss of pubic hair during chemoradiation, and chronic vaginal dryness compared with those treated with IMRT. Urinary dysfunction (frequency, dysuria, incontinence), bowel (frequency, incontinence) and sexual problems (vaginal dryness, dyspareunia, colpitis) were the main prevalent issues	8
**Mortensen (2015) **[61]	Denmark	Health-related physical, psychosexual, and social QoL and coping	Cross-sectional interview study 6–36 months post-treatment	*N* = 6 patients	RT or CRT	Qualitative interviews guided by a literature review	Patients commonly reported anogenital pain, reduced sphincter function and sexual dysfunction. Most used dissociated for maintaining normalcy: distancing from other patients with cancer, delimitation of the cancer to a perceived external and peripheral body part, and fast resumption of their usual activities	N/A (qualitative)
**Rooney (2024) **[62]	USA	Relationship between vaginal dosimetry and long-term dyspareunia post CRT	Retrospective survey. At least 2 years post-treatment. Median 58 months post-treatment	*N* = 90	CRT (IMRT)	FSFI [50] with a focus on the pain sub score (genital pain related to vaginal penetration)	Increased CRT dose to the vagina was significantly associated with worse patient-reported dyspareunia	9
Sauter (2022)* [31]	Germany	Self-reported QoL after CRT. Identification of patient-, disease-, and therapy-related factors associated with QoL	Retrospective study. Median 71 months post-treatment	*N* = 94 (*n* = 52 patients completing assessments). Comparison with a reference group of members of the German population	RT or IMRT or 3-D technique with/without CT. Surgery	QLQ-C30 [12], QLQ- ANL27 [10, 11]	Compared to the reference group, patients experienced significant problems with role, emotional, and social functioning, and with diarrhoea and constipation. Fatigue, physical, and role functioning most strongly affected global QoL causing significant psychological symptoms. Patients treated with IMRT had more problems relating to non-stoma bowel function, female sexual functioning, pain, toilet proximity and personal cleanliness compared to those treated with 3D CRT. Patients with recurrent disease and with a stoma had significantly lower bowel function. Those with relapse also experienced greater pain, stoma problems, leg oedema, needing to be near toilets, difficulty keeping cleaning and planning activities	8
**Sunesen (2021) **[63]	Denmark	Long-term symptoms and distress related to the dysfunction of pelvic organs after RT	Cross-sectional. Median 33 months post treatment	*N* = 94 eligible (*n* = 84 patients completing assessment) (no metastasis or recurrence at time of assessment and colostomy-free)	RT or CRT	Study-specific questionnaire. Urinary and sexual symptom questions were based on the LENT-SOMA [57] and anorectal symptom questions were based on the LENT-SOMA [57] questions and the St Mark’s score for faecal incontinence (Vaizey Score [56])	Anorectal, urinary, and sexual dysfunctions were common and caused significant distress. Incontinence and urgency for solid stools, liquid stools and gas occurred at least monthly in many patients (30–80%). Stress, urge or other types of urinary incontinence also occurred in 20–45% and caused great distress in 21%. Patients also had poor sexual desire and poor satisfaction	7
**Tang (2015) **[38]	USA	QoL after IMRT	Prospective. Median follow-up 3 years. Every 3 months for 2 years post-treatment and every 6 months after	*N* = 63 patients (*n* = 34 completed at least one assessment) (not metastatic)	CRT (IMRT)	FACT-C [64], MOS Sexual Problems Scale [65]	Overall quality of life scores were acceptable, but sexual functioning scores were suboptimal after IMRT. Patients also indicated problems with energy, pain, appearance, bowel control, and sexual function. Patients with a history of late gastrointestinal toxicity had worse FACT-C Physical, Functional, and Colorectal subscale scores, and patients with a history of depression or anxiety had worse Physical and Emotional subscale scores	8
**Taylor (2022) **[39]	USA	Long-term impact of CRT on bowel, sexual, and urinary function	Retrospective study with prospective data collection. Baseline, 3, 6, 12, 18, 24, months, 3 years, 4 years, and 5 years post-treatment Up to 5 years post-treatment	*N* = 143 patients	CRT	IIEF-6 erectile function domain [49], FSFI-6 [50], the OAB [66], AUA Symptom Index [67], and EQ-VAS questionnaires [40]	Major LARS at baseline was the most significant predictor of major LARS post-treatment. Poor bowel, sexual, and urinary function did not improve over time up to 2 years post treatment	8
**Yerramilli (2020) **[68]	US	Impact of CRT on sexual function, QOL, and mood	Cross-sectional. Median 36 months post treatment	*N* = 50 (*n* = 42 completed the surveys)	CRT (IMRT)	QLQ-C30 [12], QLQ-CR29 [41]. Men: IIEF [49], Women: FSFI [50], HADS [69]	Most women reported poor sexual functioning relating to satisfaction, desire, orgasm, arousal, pain, and need for lubrication and men reported poor satisfaction, erectile function, and intercourse. Despite considerable sexual function problems, patients relatively high overall QoL and low prevalence of mood symptoms One patient experienced depression, seven reported anxiety symptoms	8

*Studies include patients with metastatic and/or recurrent/persistent disease

Abbreviations:

*Condition*: LARS: Low Anterior Resection Syndrome

*Treatments:* CT: Chemotherapy, CRT: Chemoradiotherapy, External Bean Radiotherapy: EBRT, IMRT: Intensity Modulated Radiotherapy, PDR-RT: Pulsed Dose Rate Brachytherapy, RT: Radiotherapy

*Measures:* AUA American Urological Association, CTCAE: Common Terminology Criteria for Adverse Events PROM: Patient Reported Outcome Measure, EORTC: European Organisation for Research and Treatment of Cancer, QLQ-C30: Core measure; QLQ-ANL27: Anal Cancer Quality of Life Questionnaire, QLQ-CR29: Colorectal Quality of Life, EQ-5D: EuroQol Five Dimensions Questionnaire,EQ-VAS: EuroQol Visual Analogue Scale, FACT-G7: Functional Assessment of Cancer Therapy General 7 item version, FACT-C: Functional Assessment of Cancer Therapy-Colorectal Scale, FIQoL: Fecal Incontinence Quality of Life, FSFI: Female Sexual Function Index, HADS: Hospital Anxiety and Depression Scale, IBDQ-B: Inflammatory Bowel Disease Questionnaire (IBDQ), ICIQ: International Consultation on Incontinence Questionnaires, IIEF: International Index of Erectile Function, LENT-SOMA: Late Effects in Normal Tissue, Subjective Objective Management and Analytic Scales, MOS: Medical Outcomes Scale, MSKCC: Memorial Sloan-Kettering Cancer Centre Bowel Function Instrument, OAB: Overactive Bladder Questionnaire, PROMIS SexFS: Patient-Reported Outcomes Measurement Information System sexual function and satisfaction version, SOC-29: Sense of Coherence Scale

### QoL issues reported

Most studies (*n* = 16) focused on overall QoL, often with an insight into its multiple dimensions alongside clinical outcomes as part of safety assessments of treatment protocols [19, 23], to provide patients’ perspectives on outcomes [31, 36, 38, 43–45, 60, 61, 68], and as an indicator of long-term and late effects [29, 30, 33, 34, 37]. Other studies examined QoL through the lens of a particular domain such as bowel [32, 35, 39, 51, 63], urinary [35, 39, 48, 51, 63], or sexual functioning [32, 35, 47, 62, 63]. Thus, the QoL issues reported represent a function of the study’s focus and the pre-determined questions included in the PROMs used. Only three studies provided patients with the opportunity to offer an account of their experiences in their own words, either with the inclusion of a question with a free-text response to supplement the questionnaire(s) used [29, 45] or by adopting a qualitative interview design with questions informed by a literature review [61].

Across the studies, 17 PROMS were used to evaluate QoL and included one generic (non-cancer): the EuroQol Five Dimensions Questionnaire (EQ-5D) and Visual Analogue Scale (EQ-VAS) [40], and two generic cancer scales: Functional Assessment of Cancer Therapy General 7 item version (FACT-G7) [46], and the EORTC core measure (QLQ-C30) [12]. In terms of disease-specific measures, the anal cancer specific measure was used (QLQ-ANL27) [10, 11], and there were two colorectal cancer specific measures, the EORTC QLQ-CR29/38 (QLQ-29/QLQ-38) [41, 58] and the FACT-C [64]. Symptom-specific measures used included those assessing (1) bowel function, for example, the Fecal Incontinence Quality of Life (FIQoL) [52]; (2) urinary symptoms: Overactive Bladder Questionnaire (OAB) [66]; (3) sexual functioning in general, for example, PROMIS Sexual functioning [48], for males: International Index of Erectile Function (IIEF-5) [49] and for women: Female Sexual Function Index (FSFI) [50]. One study [51] used the Low Anterior Resection Syndrome Score (LARS) [53] to assess the effects of a specific condition. Three studies [30, 33, 45] created their own bespoke measures for patients to complete in order to answer their study objective and four [43, 44, 60, 63] included questions taken from existing validated measures such as the QLQ-C30 [12] and QLQ-ANL27 [10, 11].

Studies [19, 23, 43] included in this review also reported clinician rated toxicities/adverse events alongside the PROs to give additional insight into symptoms experienced by patients.

### QoL issues reported by patients with metastatic and/or recurrent/persistent anal cancer treated with systemic therapies

The two studies including patients with metastatic and/or recurrent disease present Qol scores at global [19, 23] and domain levels [19] rather than identifying particular QoL concerns. Information about specific issues facing these patients come from the reports of adverse events. Ninety-seven serious adverse events were recorded for metastatic or unresectable locally recurrent patients treated with standard or modified protocols of docetaxel, cisplatin, and fluorouracil in Kim et al.’s study [19] and included constipation, diarrhoea, nausea, vomiting, abdominal pain, and fatigue, weakness, taste changes, dehydration, mouth ulcers, peripheral neuropathy, hair loss, oedema in the limbs, hand-foot syndrome, infections, and weight loss. In Rao et al.’s study [23] of 94 patients who had been previously exposed to treatment regimens such as CRT, RT alone or surgery and currently treated with immunotherapy (500 mg of retifanlimab), 55 experienced treatment-related adverse events such as those reported by Kim et al. [19] (fatigue, nausea, diarrhoea, weakness, abdominal pain) as well as itchy skin, anaemia, and urinary tract infections.

### QoL issues reported by patients or recurrent/persistent anal cancer treated surgically

A study of patients successfully treated, on average 4 years ago earlier, with salvage surgery for recurrence [29] provided data on the EORTC core (QLQ-C30) [12] and colorectal-specific questionnaires (QLQ-CR29) [41] completed by 14 patients, as well as a list of QoL issues reported by patients which were not covered by the questionnaires. Whilst global health status, functional and symptom scores were favourable, women reported low sexual interest and problems with painful sexual intercourse, and males had high symptom scores for impotence. All symptoms measured by the QLQ-C30 [12] and the QLQ-CR29 [41] were experienced by at least one patient, most commonly fatigue (*n* = 12), dry mouth (*n* = 11), urinary frequency (*n* = 9), bloating (*n* = 9), faecal incontinence (*n* = 8), impotence (*n* = 8), and buttock pain (*n* = 7). Additional issues identified by seven patients include perineal herniation/heaviness making it difficult to sit down for long periods (*n* = 3), reduced size of penis (*n* = 1), impossibility to engage in sexual intercourse (*n* = 3), and urinary incontinence (*n* = 1).

### Long-term QoL issues of patients treated for anal cancer (not specifically for metastatic or recurrent disease)

As mentioned above, the three main QoL domains assessed in studies of long-term and late effects of patients previously treated for anal cancer include bowel, urinary, and sexual functioning, with the data showing problems in each of these areas persisting over time (as long as 6 years post-diagnosis) and subsequently impacting perceptions of overall QoL (e.g. [30, 38]. In addition, long-term effects of bowel habit changes, sexual function, and interest were reported as worse than expected by over half patients in a study by Chaballout et al. [44]. In terms of bowel functioning, the most prevalent long-term issues (experienced at least 12 months post-treatment) include diarrhoea [31, 35, 36, 63], constipation [31], faecal incontinence or leakage [35, 37, 38, 60, 63], urgency [35, 63], frequency [60], need to be close to the toilet [31], difficulties cleaning oneself [31], and flatulence [35, 63]. Urinary problems include bladder incontinence [36, 37, 60, 63], frequent urination [32, 60] and painful urination [60]. Sexual problems reported include painful sexual intercourse [36, 37, 60, 62, 68], low sexual desire and interest [63, 68], vaginal dryness [60, 68], vaginal inflammation [60], and impotence [34, 68].

Other long-term issues reported include insomnia [35], low energy [38], weakness [43], breathlessness [34], pain [38, 43], body image problems [34, 38], embarrassment [34, 45], financial difficulties amongst patients < 65 years [35], hair loss [34], and diet and activity limitations [31, 35, 43].

Studies provided evidence not only of persistence in problems but also progressively worsening of bowel and sexual function problems over time with a peak in symptom prevalence between 2 and 5 years post-diagnosis reported by Frick et al. [33], although separate data for patients with anal cancer were not provided for their change over time analysis. Hosni et al. [34] reported long-term decline in breathlessness, body image, bowel embarrassment, faecal incontinence, and hair loss, and a worsening of impotence.

In terms of free text responses to questions inviting patients to share additional thoughts on how anal cancer and its treatment had affected them, including issues which had not been addressed in the questionnaires, persistent or permanent late toxicity from CRT was reported by 82% patients included in a study by Corrigan et al. [45]. These issues include bowel (diarrhoea, incontinence, flatulence), urinary (frequency), sexual (avoidance of intercourse, fear of pain) and musculoskeletal (hip pain, bone fractures, loss of mobility impacting daily activities and ability to exercise) problems. Patients interviewed by Mortensen and Lundby [61] also described anogenital pain, reduced sphincter function, and sexual dysfunction.

Table 4 summarises the full list of the 53 QoL issues extracted from the studies involving patients with metastatic and recurrent/persistent anal cancer treated with systemic therapies (*n* = 17 issues), recurrence treated with surgery (*n* = 11 issues), and those presenting long-term follow-up data on previously treated patients (*n* = 31 issues). Only seven QoL issues were reported in both studies involving patients with metastatic or recurrent anal cancer and those involving disease-free survivors. Twenty issues, including neuropathy and skin and mouth problems, were uniquely reported in studies involving patients with metastatic or recurrent disease, 13 of which were only reported by those patients receiving systemic treatments of CT or immunotherapy. Whilst bowel and sexual problems were reported by patients with metastatic and recurrent/persistent disease, these were particularly prominent as late effects of CRT.

**Table 4:** QoL issues extracted from the studies included in the review

QoL issue	Reported by patients with metastatic/recurrent anal cancer treated with systemic therapies	Reported by patients treated with surgery for recurrence	Reported as a long-term and late effect of anal cancer treatment (CRT)	Included within the QLQ-C30 or QLQ-ANL27
**Bowel function**				
Constipation	X			X
Diarrhoea	X		X	X
Faecal incontinence		X	X	X
Urgent bowel movements			X	X
Frequent bowel movements			X	X
Need to be close to the toilet			X	X
Difficulty cleaning oneself				X
Stoma problems				X
Flatulence			X	X
**Urinary function**				
Urinary tract infections	X			
Frequent urination		X	X	X
Urinary incontinence		X	X	
Painful urination			X	
**Sexual function**				
Inability to engage in sexual intercourse		X		X
Avoidance of sexual intercourse			X	X
Painful sexual intercourse			X	X
Low sexual desire or interest			X	X
Vaginal dryness			X	X
Vaginal inflammation			X	
Impotence		X	X	X
Reduction in size of penis		X		
**Emotional function**				
Body image			X	
Embarrassment			X	
Sadness			X	X
**Pain**				
Anogenital pain			X	X
Buttock pain		X		X
Abdominal pain	X			
Hip pain			X	
Pain (site not specified)			X	X
**Gastro-intestinal problems** (other than bowel function)				
Nausea	X			X
Vomiting	X			X
Abdominal bloating		X		
Diet restrictions			X	
**Skin and hair problems**				
Hand-foot syndrome	X			
Itchy skin	X			X
Hair loss	X		X	
**Mouth problems**				
Taste changes	X			
Dry mouth		X		
Dehydration	X			
Mouth ulcers	X			
**Other QoL concerns**				
Low energy			X	
Insomnia			X	X
Weakness	X		X	X
Breathlessness			X	X
Difficulty sitting for long periods		X		X
Weight loss	X			
Peripheral neuropathy	X			
Fluid retention in the limbs	X			X
Loss of mobility			X	X
Bone fractures (pelvic region)			X	
Restricted activities			X	X
Financial difficulties			X	X

### Mapping of QoL issues against the QLQ-C30 and QLQ-ANL27

Table 4 also maps the issues extracted from the studies onto questions included in the core EORTC and anal-cancer specific QoL measures. The EORTC measures include 32 of the 53 issues captured. Two issues missing in the measures, hair loss and urinary incontinence, were reported in studies including metastatic, recurrent, and disease-free patients. Eleven missing issues were extracted from studies including patients with metastatic and/or recurrence including abdominal pain, abdominal bloating, weight loss, taste changes, dry mouth, dehydration, mouth ulcers, peripheral neuropathy, hand-foot syndrome, infections, and reduced size of penis. Eight long-term issues reported in the literature of disease-free survivors and not covered by the measures include low energy, diet restrictions, painful urination, vaginal inflammation, body image, embarrassment, bone fractures, and hip pain.

## Discussion

This systematic review of 23 publications reported QoL concerns of patients treated for anal cancer with a specific focus on patients with metastatic and/or recurrent/persistent disease. There was a paucity of data on the specific QoL issues experienced by the patient group of interest with only three papers exclusively focusing on patients with metastatic and/or recurrent/persistent disease, with the remaining 20 providing data on long-term effects of patients who were mainly treated for CRT for anal cancer. Five of these studies reported that patients with metastatic and/or recurrent disease were included in their cohort although the numbers of such patients and their specific QoL concerns were not always clearly identified. It is also possible that more of the studies included in this review reported data on patients who had developed metastases or recurrence during follow-up, but this was not clear in our data extraction process. Rather than restricting the inclusion to the three papers focusing only on patients with metastatic and/or recurrent disease, we felt there was merit in evaluating the long-term data of patients previously treated with CRT for anal cancer, given that patients experiencing disease progression or recurrence have previously been exposed to these treatments and their late effects which consequently adds to the burden of their current treatments.

An exhaustive list of 53 issues was established from the literature on QoL of patients and included 17 issues from the two studies [19, 23] evaluating the safety of systemic therapies including docetaxel, cisplatin, and fluorouracil [19] and retifanlimab [23]. Although patients completed QoL questionnaires in these studies, information about specific QoL issues was extracted from data on adverse events and covered fatigue, weakness, gastro-intestinal and skin problems, hair loss, peripheral neuropathy symptoms, and fluid retention. A total of 11 issues were extracted from the study on patients previously treated with salvage surgery for recurrence [29] and highlighted persistent problems with bowel, urinary, and sexual functioning, a finding which was echoed in the studies which focused on the late and long-term effects of treatment for anal cancer and was documented in our earlier systematic review [5]. Whilst there was overlap in QoL issues across the different disease (metastatic, recurrent or long-term follow up disease free) and treatment types (systemic or surgery), some issues, such as neuropathy and skin and mouth problems, were only reported in the studies involving patients in the metastatic or recurrent setting.

Several of the measures used to evaluate QoL in the studies covered questions about bowel, urinary, and sexual functioning in these areas; therefore, it is not surprising that issues relating to bowel and urinary problems (i.e. frequency, urgency, incontinence) as well as sexual functioning (i.e. interest, pain, impotence) are reported as the most prevalent in this review. The EORTC QoL questionnaires were the most frequently used across the studies, with nine using the core questionnaire (QLQ-C30) [12] which was supplemented with the disease specific measure for colorectal cancer (QLQ-CR29) [41] in six studies and for anal cancer (QLQ-ANL27) [10, 11] in two studies, although the data for the QLQ-ANL27 were only presented in one of these studies [31]. In addition, some of the items from the EORTC QoL questionnaires (including the QLQ-ANL27) were selected to form bespoke study-specific measures used in two studies.

Three studies were less prescriptive in the QoL issues captured by providing patients with the opportunity to describe their experiences in their own words and went beyond the three main domains described above. For example, in Corrigan et al.’s [45] thematic analysis of patients’ experiences, persistent toxicities also included musculoskeletal problems (bone fractures, hip pain and subsequent impaired mobility) which were not reported in any of the other studies included in this review.

Out of the 53 issues captured, over half (*n* = 32) were covered by either the QLQ-C30 or the QLQ-ANL27 which again is perhaps not surprising given that a lot of the data included in this review represent scores from the EORTC QoL measures. Issues covered by the QLQ-ANL27 were informed by our original literature review, extensive interviews with patients with anal cancer, and health care professionals treating anal cancer internationally. Questionnaire items were then formulated based on pre-existing items within the EORTC item library [26] many of which (e.g. stoma care and bowel function) were part of the pre-existing colorectal-specific questionnaire (QLQ-CR29) [41]. Of particular interest, for the purposes of evaluating whether the QLQ-ANL27 is suitable for use with patients with metastatic or recurrent/persistent anal cancer, are the novel issues captured from the studies. The following additional issues might also need to be considered in future evaluations of QoL for these patients: urinary incontinence, abdominal pain and bloating, weight loss, taste changes, dry mouth, dehydration, mouth ulcers, hand-foot syndrome, hair loss, peripheral neuropathy, change in genitalia, and infections. Additional issues extracted from studies on long-term survivors and not covered by the EORTC core or anal cancer measures include low energy, diet restrictions, painful urination, body image, embarrassment, bone fractures, and hip pain. It is not clear the extent to which these issues also represent QoL issues of patients with metastatic or recurrent/persistent disease or whether they are experienced only by those treated with CRT for localised disease. Although this review represents an extension of our previous systematic review of the QoL issues of patients with anal cancer with a refined focus on metastatic and recurrent disease, the findings, especially with respect to bowel, urinary, and sexual function, overlap with those from our previous systematic literature review, as well as a more recent review by Sterner et al. [70]. Several of the issues captured from our earlier review and in the current review, such as urinary frequency, embarrassment, and bone fractures were considered for inclusion in the QLQ-ANL27 but were not included in the final validated version following its rigorous and robust testing.

### Limitations

A limitation of this systematic review is the lack of data available on outcomes of patients with metastatic or recurrent/persistent anal cancer as described or rated by the patients themselves. Only two studies involving such patients who were currently receiving systemic treatment met the inclusion criteria and a further study involved reported data from patients who had been successfully treated with salvage therapy for recurrence. Therefore, our ability to provide an exhaustive list of QoL issues experienced by our patient group of interest was limited. The list of issues extracted in this review was also informed by studies reporting long-term QoL outcomes of patients, with several of these studies being retrospective in nature. It could however be argued that the list was contaminated to some extent by the inclusion of short-term issues/acute effects which might not be relevant to patients with metastases or recurrence. Care was taken in this review to focus only on the long-term and late effects. In the five studies reporting long-term QoL outcomes of patients and which reported the inclusion of patients, either presenting with metastases or recurrence or experiencing treatment failure during the course of the study, the numbers were not always clearly stated and QoL data specific to these patients were not presented separately. Given the complex treatment history of some of the patients included in this review, it was also not possible to make any assertions regarding causality, i.e. whether QoL issues reported were the result of the disease itself, current systemic therapies, surgery, or previous CRT.

This systematic review included studies which presented patient reported QoL. However, for the two studies involving patients treated systemically for metastases or recurrence, adverse event reporting was informative in terms of potential QoL issues, in lieu of patient reported QoL scores reported as a total rather than individual or domain symptom and functioning scores. It could be argued that this review should have broadened its inclusion criteria to include studies reporting only clinician-rated toxicities. Several papers reporting trials of new systemic treatments for patients with metastatic and/or recurrent disease (i.e. [14, 21, 22]) were not eligible because of the absence of PROs.

In addition, whilst the focus of the review is on the data captured from PROs, there was a reliance upon measures with their ‘pre-determined’ issues and, except for one qualitative study and two which included a free-text response option, there was a limited opportunity for patients to report experiences not covered by existing measures. Therefore, the QoL issues reported were often a function of the QoL measures used.

Finally, not only was there a paucity of studies involving patients with metastatic or recurrence, but the sample sizes were low, for example only six patients interviewed by Mortensen and Lundby [61] and 22 patients completing questionnaires in a study by Pederson et al. [29]. Given that anal cancer is rare, it is perhaps not surprising that low numbers of patients are recruited to studies but nonetheless power is compromised. In addition, the number of patients completing assessments out of the total number of eligible is often below 50% and high levels of attrition are noted. This review underlines the need for additional studies focusing on the specific QoL concerns of patients with metastases or recurrence as reported by the patients themselves. Moreover, the need for the development of a strategy for assessing the QoL of such patients is also highlighted.

## Conclusion

Whilst there is a growing body of evidence of patient reported QoL concerns in patients with anal cancer, it is predominantly limited to those being treated with CRT for curative intent. However, patients with metastatic or recurrent disease, who are potentially already challenged by the legacy of an intensive course of CRT before starting further treatment protocols of systemic therapies or surgery, are likely to experience complex and unique concerns. To our knowledge, this is the first systematic review to devote attention to the QoL concerns of such patients with the intention of generating an exhaustive list of QoL issues. Whilst most issues captured are covered by the only QoL questionnaire specific to patients with anal cancer, QLQ-ANL27, used alongside the core cancer measure, the QLQ-C30, some concerns appeared to be overlooked and need to be further explored in future research with this under-researched patient group to include patient and health care professional interviews.

## Supplementary information

Below is the link to the electronic supplementary material.

ESM 1 (DOCX 86.3 KB)

## Data Availability

No datasets were generated or analysed during the current study.
