# Supplementary material for: Improving our understanding of the quality of life of patients with metastatic or recurrent/persistent anal cancer: a systematic review
Source: Support Care Cancer. 2025 May 15;33(6):475. doi: 10.1007/s00520-025-09520-8 (PMC12081495; doi:10.1007/s00520-025-09520-8)
Supplement: Supplementary file 1 — (DOCX 86.3 KB) [file 520_2025_9520_MOESM1_ESM.docx]

**A systematic review of Quality of Life (QoL) issues in patients with metastatic or recurrent/ persistent anal cancer**

**Review Protocol**

**Version 1.0 Date: 01/11/2023**

***The protocol has been written on behalf of the Module Development Team for the Phase I/II feasibility study to develop a HRQoL module for metastatic and recurrent/ persistent anal cancer or to add metastatic anal cancer-specific items to the existing EORTC QLQ-ANL27***

Review Team

| **Name** | **Affiliation** | **E-mail** | **Role and Contribution** |
| --- | --- | --- | --- |
| Rowan Edwards  (RE) | University of Southampton, UK | [R.J.R.Edwards@soton.ac.uk](mailto:D.Fitzsimmons@swansea.ac.uk) | **Study coordinator**  Conceptualised and drafted the protocol.  Will perform searches, screen and co-review articles.  Will undertake data extraction, analysis and write up. |
| Samantha Sodergren  (SS) | University of Southampton, UK | [S.C.Sodergren@soton.ac.uk](mailto:S.C.Sodergren@soton.ac.uk) | **Principal Investigator**  Will review protocol.  Will screen and co-review articles.  Will review data extraction  Will support the analysis and write up. |
| Vassilios Vassiliou  (VV) | Bank of Cyprus Oncology Centre, Nicosia, Cyprus | [vasilis.vasiliou@bococ.org.cy](mailto:vasilis.vasiliou@bococ.org.cy). | **Principal Investigator**  Will review protocol.  Will support the analysis and write up. |
| Kristopher Dennis  (KD) | The Ottawa Hospital Research Institute, Canada | krdennis@toh.ca | **Module development team collaborator.**  Will review protocol.  Will co-review articles.  Will support the analysis and write up. |
| Pierfrancesco Franco  (PF) | University of Eastern Piedmont Hospital, Italy | pierfrancesco.franco@uniupo.it | **Module development team collaborator.**  Will review protocol.  Will co-review articles.  Will support the analysis and write up. |
| Marianne Grønlie Guren  (MG) | Oslo University Hospital, Norway | Marianne.Gronlie.Guren@ous-hf.no | **Module development team collaborator.**  Will review protocol.  Will co-review articles.  Will support the analysis and write up. |
| Said Al Saifi  (SA) | The Ottawa Hospital Research Institute, Canada | salsaifi@toh.ca | **Module development team collaborator.**  Will review protocol.  Will co-review articles.  Will support the analysis and write up. |
| Francesca De Felice  (FDF) | Sapienza University of Rome, Italy | [francesca.defelice@uniroma1.it](mailto:francesca.defelice@uniroma1.it) | **Module development team collaborator.**  Will review protocol.  Will support the analysis and write up. |

**Corresponding author:** Dr Rowan Edwards, School of Health Sciences, Building 67, University of Southampton, Highfield, Southampton, SO17 1BJ.

[R.J.R.Edwards@soton.ac.uk](mailto:D.Fitzsimmons@swansea.ac.uk)

**Funder and Sponsor**

**Financial Support:** This review is funded by the EORTC Quality of Life Group as part of a grant award (ref. 006-2022) for the Phase I/II feasibility study to develop a HRQoL module for metastatic and recurrent/ persistent anal cancer or to add metastatic anal cancer-specific items to the existing EORTC QLQ-ANL27

1. **Sponsor:** The overall sponsor for the above- mentioned award is University of Southampton, UK
2. **Role of funder/ sponsor in protocol development.**

The funder or sponsor have had no direct input into the development of the protocol.

**Title**

A systematic review of Quality of Life (QoL) issues for patients with metastatic or recurrent/ persistent anal cancer

**Background and rationale**

**Patient-reported outcomes in oncology**

In oncology in the last two decades, there has been growing awareness of the importance to complement the traditional clinician-rated assessment of the patient’s health status with an assessment made by the patients themselves i.e., patient-reported outcomes (PROMs). According to Basch [[1](#_ENREF_1)], PROM data improves the detection of adverse events in clinical trials as including only clinician’s ratings is known to lack sensitivity. This means that worrisome symptoms might well come to light earlier in the drug-development cycle if reporting by patients is standard practice. Therefore, adding PROMs to clinical trials may lead to a major improvement of symptom management in oncology research.

Consequently, the assessment of health-related quality of life (HRQoL) is now a widely used parameter in clinical oncology research, and over the last 15 years an important issue in the area has been to establish valid and reliable instruments to assess HRQoL of cancer patients [[2](#_ENREF_2)]. In this context, HRQoL is often used as an umbrella term to refer to certain aspects of functioning (e.g. physical, emotional, social, role) and major symptoms (e.g. pain, fatigue, gastrointestinal symptoms). As a result of such research, several cancer-specific HRQoL instruments have been developed, including the EORTC QLQ-C30 developed by the European Organization for Research and Treatment of Cancer (EORTC) Quality of Life Group (QLG) [[3](#_ENREF_3)].

**Metastatic or recurrent/ persistent anal cancer**

Anal carcinomas arise at the anal margin or within the anal canal with the vast majority being squamous cell carcinomas. These carcinomas are rare, accounting for less than 3.5 % of all GI cancers [[4-7](#_ENREF_4)]. However, the incidence is increasing worldwide. The current standard of care is definitive chemoradiotherapy (CRT), achieving 5-year survival rates of 75% [[8](#_ENREF_8)]. Even though CRT is effective, it is associated with acute and chronic toxicity/complications that impair the health-related quality of life (HRQoL) of treated patients [[9](#_ENREF_9)], both during and post treatment. HRQoL of individuals treated with CRT for anal cancer can be assessed using a validated disease specific tool, the EORTC QLQ-ANL27 (QLQ-ANL27) [[10](#_ENREF_10)] module. The QLQ-ANL27 is the only HRQoL measure specific to patients with anal cancer who have been treated with CRT. The extent to which the module is applicable to patients with metastatic anal cancer or with persistent or recurrent anal cancer is not yet known; the Phase IV validation study included 6 patients with metastatic cancer, 8 with local recurrence and 7 with locoregional recurrence. In addition, since the work on this module began, the treatment landscape for anal cancer has changed to include novel treatments such as biological / targeted therapies and immunotherapies.

About 15% of anal cancer patients are diagnosed with advanced disease and 15-20% will develop diseases progression after CRT [[11](#_ENREF_11)]. In a recent study of 132 patients with anal cancer, 14% experienced locoregional and/or distant treatment failure during a median follow-up period of 49.3 months [[12](#_ENREF_12)]. In cases with localised operable recurrence patients may undergo salvage surgery such as abdominoperineal resection. In cases with distant failure or inoperable locoregional disease, patients are managed with chemotherapy regimens [[13](#_ENREF_13)]. Fluorouracil and cisplatin have previously been mostly used, however a multi-centre randomized trial recently determined carboplatin and paclitaxel as the preferred chemotherapy regimen [[11](#_ENREF_11)]. The combination of docetaxel, cisplatin, and fluorouracil has also shown efficacy in a single-arm phase 2 study [[14](#_ENREF_14)]. More recently treatment with checkpoint inhibition such as with nivolumab, pembrolizumab and retifanlimab have shown effectiveness and are used in clinical trials [[15-17](#_ENREF_15)], also in combination with chemotherapy, such as the ongoing SCARCE GERCOR trial with atezolizumab [[18](#_ENREF_18)], as well as being used concurrently with radiotherapy for managing localized anal cancer in clinical trials [[19](#_ENREF_19)]. While the advent of immune therapies are welcomed as a promising treatment of advanced anal cancer these therapies are delivered over a longer period and are likely to have other immune-related side effect profiles which might not covered by existing measures including the QLQ-ANL27. As well as managing treatment side effects, patients with metastatic anal cancer might also find themselves dealing with localised recurrence, and the late effects of CRT and of extensive salvage surgery.

It is hypothesised that while the QLQ-ANL27 is likely to capture several concerns experienced in the metastatic or recurrent/ persistent context, the QLQ-ANL27 might need to be supplemented with additional items. The EORTC QLG supports this flexible approach and has an item library which can be used to supplement existing modules and for the creation of bespoke, customised lists [[20](#_ENREF_20)]. Therefore, this review focuses on identifying health related quality of life (HRQoL) issues specific to the group of patients with metastatic and recurrent/persistent anal cancer.

## **EORTC guidelines for Module Development**

The EORTC QLG have published guidelines for the development of HRQoL (PRO) questionnaire modules for the use in international cancer clinical trials and studies [[21](#_ENREF_21)]. A literature review forms part of the Phase I work which helps to inform the content of subsequent interviews with patients and health care professionals.

**Review Question**

What are the HRQoL issues for patients diagnosed with metastatic or recurrent/ persistent anal cancer?

**Review Objectives**

1. To identify the HRQoL issues relevant to anal cancer patients with a metastatic, recurrent, or persistent diagnosis. The review will consider HRQoL concerns at the time of diagnosis, during treatment and following treatment.
2. To capture the broadest range of issues, the review will incorporate patient-reported experiences of long-term and late effects of previous treatments and surgeries, which have the potential to affect individuals living with metastatic anal cancer or with recurrence/ persistence.

Table 1: Review PICO

| **Population** | Individuals aged 18 years and older with a diagnosis of metastatic or recurrent/ persistent anal cancer |
| --- | --- |
| **Intervention** | *Treatment options available to this patient group, including:*  CRT  Chemotherapy  Combination chemotherapy  Combined treatment (i.e., chemotherapy plus targeted therapy) Approved immunotherapy  Salvage surgery |
| **Comparison** | Not applicable |
| **Outcome(s)**  ***Objective*** | All issues under HRQoL domains:   - Symptoms - Treatment side-effects (acute/long-term) - Physical - Psychological - Social/Role - Cognitive - Financial - Sexual health - General health/Quality of Life |

**Methods of Review**

A systematic review will be undertaken. The protocol structure and content has been informed by the PRISMA-P statement [[22](#_ENREF_22)] on reporting and developing a systematic review protocol. The protocol also considers the COSMIN methodology for systematic reviews of patient reported outcomes [[23](#_ENREF_23)].

**Setting**

Literature will be considered from all clinical settings (e.g., primary care, secondary care, palliative care) and countries.

**Time frame**

The review will search literature that has been published since the initial systematic literature review for anal cancer [[9](#_ENREF_9)].

- Start date: 01 April 2014
- End date: 16 May 2023

**Language**

English

##

**Publication status**

All available peer-reviewed literature will be considered. Reports of conference proceedings, theses, abstracts, and case reports will be excluded.

In order to capture any publications made available in the months following the first search, a second search, exactly mirroring the first search will be conducted prior to writing up.

**Eligibility criteria**

The focus is on primary studies that report potential HRQoL concerns for patients diagnosed with metastatic or recurrent/ persistent anal cancer.

- Primary research studies publishing patient-reported HRQoL concerns/issues for this patient group.
- Studies which report the experience of metastatic or recurrent/ persistent anal cancer from the patient’s perspective.
- Studies which report the late effects of all treatments for anal cancer from the patient’s perspective.

***Unless the anal cancer patients are reported in a clearly defined separate group, publications which include patients with anal cancer alongside other patient groups will be excluded.***

**Search strategy**

As this literature review is an extension of the initial work undertaken for the EORTC QLQ-ANL27 [[9](#_ENREF_9)], the same search strategy will be adopted. This strategy has been previously reviewed by the original research team and verified by the University of Southampton library specialists.

The search process and results will be recorded simultaneously. References will be managed using Endnote and Rayyan. For electronic database searches, the following information will be recorded:

- The name of the database searched
- The date when the search was run
- The years covered by the search
- The full strategy used

**Bibliographic databases**

The search will be made through the University of Southampton library bibliographic databases for:

- [MEDLINE](https://search.ebscohost.com/login.aspx?authtype=shib&direct=true&db=cmedm&custid=s8000044)
- [Psych Info](http://search.ebscohost.com/login.aspx?authtype=shib&custid=s8000044&profile=ehost&defaultdb=psyh)
- [Web of Science](https://ifind.swan.ac.uk/view/action/uresolver.do?operation=resolveService&package_service_id=36877277100002417&institutionId=2417&customerId=2415)
- [CINAHL](http://search.ebscohost.com/login.aspx?authtype=shib&direct=true&db=rzh&custid=s8000044)
- [EMBASE](https://www.embase.com/landing?status=grey)

and the Cochrane Database of Systematic Reviews (CDSR)

**Other sources**

Reference lists from articles eligible for inclusion will also be hand searched to identify additional studies. Where applicable, related searches and author searches will be used in electronic databases to locate other relevant papers.

**Search Terms**

The search terms used in this review had been previously developed during the original literature review on HRQoL and anal cancer [[9](#_ENREF_9)]. Search expressions will be created using the Boolean operator OR between each term in each area and the Boolean operator AND between each area.

It was decided to maintain broader terms referring to anal cancer rather than specific ‘metastatic’, ‘recurrent’ or ‘persistent’ terms as 1) late and lasting effects of previous anal cancer treatments will affect this patient group and 2) some publications may refer to this latter patient group within a broader report on all patients with anal cancer.

Table 2: Search terms applied

| **Area** | **Terms** |
| --- | --- |
| Anal Cancer | Anus neoplasm (MeSH term)  Anal neoplasm  Anal cancer  Anus cancer  Anal carcinoma  Anus carcinoma (no hits)  Anal canal cancer  Anal canal carcinoma  Anal tumour  Anus tumour (no hits)  Anal intraepithelial neoplasia  Anal canal intraepithelial neoplasia  Anal squamous intraepithelial lesions  Anal squamous cell carcinoma  Anal cloacogenic carcinoma (no hits)  Cloacogenic carcinoma of the anal canal |
| Treatments  Radiochemotherapy  Stoma | Chemoradiotherapy  Radiochemotherapy  Chemoradiation  Chemotherapy  Radiotherapy  Combined modality therapy  Antineoplastic chemotherapy  Antineoplastic agents  Colostomy  Surgical stoma (Exp Stoma and stoma bag) |
| Health-related quality of life | Quality of Life  QOL  Health related quality of life  HRQOL  Subjective health status  Patient reported outcome  Patient based outcome  Patient reported outcome measure  PROM  Self report  Side effect  Toxicity  Adverse effect  Adverse event  Safety  Complication  Dysfunction  Disturbance  Disorder  Impairment  Complaint  Symptom |

## **Selection process**

**Stage one**

Based on titles, duplicated records will be removed using the Endnote facility and will also be cross-checked. Two reviewers (RE and SS) will independently screen the titles/abstracts of all identified hits against the eligibility criteria. Titles and abstracts will be assessed against the inclusion criteria with a bias towards over-inclusion.

**Stage two**

For studies that appear to meet the inclusion criteria or cannot be rejected on the basis of title and/or abstract alone, the full paper will be obtained and then checked against the inclusion criteria. If the amount of information reported about a study is insufficient to decide about inclusion, authors will be contacted. If the authors fail to respond after a two-week interval, a second attempt at contact will be made. In situations where authors do not reply after a further two weeks, the study will be listed as “potentially relevant”.

The eligibility of selected papers will be assessed by two researchers using Rayyan software (RE will assess all articles and SS, MG, KD, PF and SA will assess a quota). Disagreements will be resolved by discussion if possible, and then by consultation with a third researcher if necessary.

Data will be extracted onto a standard template on an Excel spreadsheet. This will be done by one reviewer (RE), with a 10% random sample checked for final inclusion and data extraction by a second reviewer (SS).

A PRISMA flowchart will be populated to document the results of the search and selection process.

**Data collection**

For all included publications, the following will be summarised (but not be limited to):

- Author and Year
- Title
- Publication
- Study type
- Participants: numbers and characteristics (including type of diagnosis and stage of disease)
- Treatments
- Context (country and healthcare setting)
- Aim of study
- Data collection methods
- Data collection timepoints
- Data analysis methods
- Theoretical models adopted for interpretation/contextualisation (if applicable)
- HRQoL issues reported
- HRQoL domains assessed (e.g., symptoms, physical functioning)
- Analysis of outcomes
- Key findings
- Conclusion and comments

Where reviews, reports or meta-analyses are identified these will be considered for descriptive and cross-referencing purposes only but will not be formally used for final inclusion/data extraction to avoid duplication.

**Data Synthesis**

Due to the heterogeneity of studies in relation to research focus, treatments assessed, measures used and time of assessment, a descriptive synthesis of the data will be used.

**References**

1. Basch, E., *The missing voice of patients in drug-safety reporting.* New England Journal of Medicine, 2010. **362**(10): p. 865-869.

2. Fayers, P.M. and D. Machin, *Quality of life: the assessment, analysis and interpretation of patient-reported outcomes*. 2013: John Wiley & Sons.

3. Aaronson, N.K., et al., *The European Organization for Research and Treatment of Cancer QLQ-C30: a quality-of-life instrument for use in international clinical trials in oncology.* JNCI: Journal of the National Cancer Institute, 1993. **85**(5): p. 365-376.

4. Islami, F., et al., *International trends in anal cancer incidence rates.* International journal of epidemiology, 2017. **46**(3): p. 924-938.

5. Ries, L.A., et al., *SEER cancer statistics review, 1975-2003.* 2006.

6. Siegel, R.L., K.D. Miller, and A. Jemal, *Cancer statistics, 2018.* CA: a cancer journal for clinicians, 2018. **68**(1): p. 7-30.

7. Uronis, H.E. and J.C. Bendell, *Anal cancer: an overview.* The Oncologist, 2007. **12**(5): p. 524-534.

8. Sekhar, H., et al., *Temporal improvements in loco-regional failure and survival in patients with anal cancer treated with chemo-radiotherapy: treatment cohort study (1990–2014).* British Journal of Cancer, 2020. **122**(6): p. 749-758.

9. Sodergren, S.C., et al., *Systematic review of the quality of life issues associated with anal cancer and its treatment with radiochemotherapy.* Supportive Care in Cancer, 2015. **23**: p. 3613-3623.

10. Sodergren, S.C., et al., *Phase I–III development of the EORTC QLQ-ANL27, a health-related quality of life questionnaire for anal cancer.* Radiotherapy and Oncology, 2018. **126**(2): p. 222-228.

11. Rao, S., et al., *International rare cancers initiative multicenter randomized phase II trial of cisplatin and fluorouracil versus carboplatin and paclitaxel in advanced anal cancer: InterAAct.* Journal of Clinical Oncology, 2020. **38**(22): p. 2510.

12. Slørdahl, K.S., et al., *Treatment outcomes and prognostic factors after chemoradiotherapy for anal cancer.* Acta Oncologica, 2021. **60**(7): p. 921-930.

13. Sclafani, F. and S. Rao, *Systemic therapies for advanced squamous cell anal cancer.* Current oncology reports, 2018. **20**: p. 1-11.

14. Kim, S., et al., *Docetaxel, cisplatin, and fluorouracil chemotherapy for metastatic or unresectable locally recurrent anal squamous cell carcinoma (Epitopes-HPV02): a multicentre, single-arm, phase 2 study.* The Lancet Oncology, 2018. **19**(8): p. 1094-1106.

15. Morris, V.K., et al., *Nivolumab for previously treated unresectable metastatic anal cancer (NCI9673): a multicentre, single-arm, phase 2 study.* The Lancet Oncology, 2017. **18**(4): p. 446-453.

16. Ott, P., et al., *Safety and antitumor activity of the anti-PD-1 antibody pembrolizumab in patients with recurrent carcinoma of the anal canal.* Annals of Oncology, 2017. **28**(5): p. 1036-1041.

17. Rao, S., et al., *A phase II study of retifanlimab (INCMGA00012) in patients with squamous carcinoma of the anal canal who have progressed following platinum-based chemotherapy (POD1UM-202).* ESMO open, 2022. **7**(4): p. 100529.

18. Stefano K, F.G., Jerome D, et a, *Atezolizumab plus modified*

*docetaxelcisplatin-5-fluorouracil (mDCF) regimen versus mDCF in patients*

*with metastatic or unresectable locally advanced recurrent anal*

*squamous cell carcinoma: a randomized, non-comparative phase II SCARCE*

*GERCOR trial*. 2020. p. 352.

19. Martin, D., et al., *RADIANCE–Radiochemotherapy with or without Durvalumab in the treatment of anal squamous cell carcinoma: A randomized multicenter phase II trial.* Clinical and translational radiation oncology, 2020. **23**: p. 43-49.

20. Kulis, D., et al., *The use of the EORTC item library to supplement EORTC quality of life instruments.* Value in Health, 2017. **20**(9): p. A775.

21. Bjordal K, B.A., Gilbert A, Martinelli F, Pe M, Sztankay M, et al. , *EORTC Quality of Life Group Module Development Guidelines (5th Edition)*. 2021.

22. Page, M.J., et al., *The PRISMA 2020 statement: an updated guideline for reporting systematic reviews.* International journal of surgery, 2021. **88**: p. 105906.

23. Mokkink, L.B., et al., *COSMIN methodology for systematic reviews of patient-reported outcome measures (PROMs).* User manual, 2018. **78**(1): p. 6-3.
